# The Association of Factor V Leiden and Prothrombin Gene Mutation and Placenta-Mediated Pregnancy Complications: A Systematic Review and Meta-analysis of Prospective Cohort Studies

**DOI:** 10.1371/journal.pmed.1000292

**Published:** 2010-06-15

**Authors:** Marc A. Rodger, Marisol T. Betancourt, Peter Clark, Pelle G. Lindqvist, Donna Dizon-Townson, Joanne Said, Uri Seligsohn, Marc Carrier, Ophira Salomon, Ian A. Greer

**Affiliations:** 1 Thrombosis Program, Division of Hematology, Departments of Medicine, Obstetrics and Gynecology and Epidemiology/Community Medicine, University of Ottawa, Ottawa, Ontario, Canada; 2 Clinical Epidemiology Program, Ottawa Hospital Research Institute, The Ottawa Hospital, Ottawa, Ontario, Canada; 3 Department of Transfusion Medicine, Ninewells, Hospital and Medical School, Dundee, United Kingdom; 4 Department of Obstetrics and Gynecology, Karolinska Hospital, Huddinge, Sweden; 5 National Institute of Child Health and Human Development Maternal-Fetal Medicine Units Network, Bethesda, Maryland; 6 Department of Perinatal Medicine, The Royal Women's Hospital, Carlton, Victoria Australia; 7 Department of Obstetrics and Gynaecology, The University of Melbourne, Parkville, Victoria, Australia; 8 The Amalia Biron Thrombosis and Hemostasis Research Institute, the Chaim Sheba Medical Center, Tel Hashomer, Israel; 9 Hull York Medical School, York, United Kingdom; University of Queensland Centre for Clinical Research, Australia

## Abstract

**Background:**

Factor V Leiden (FVL) and prothrombin gene mutation (PGM) are common inherited thrombophilias. Retrospective studies variably suggest a link between maternal FVL/PGM and placenta-mediated pregnancy complications including pregnancy loss, small for gestational age, pre-eclampsia and placental abruption. Prospective cohort studies provide a superior methodologic design but require larger sample sizes to detect important effects. We undertook a systematic review and a meta-analysis of prospective cohort studies to estimate the association of maternal FVL or PGM carrier status and placenta-mediated pregnancy complications.

**Methods and Findings:**

A comprehensive search strategy was run in Medline and Embase. Inclusion criteria were: (1) prospective cohort design; (2) clearly defined outcomes including one of the following: pregnancy loss, small for gestational age, pre-eclampsia or placental abruption; (3) maternal FVL or PGM carrier status; (4) sufficient data for calculation of odds ratios (ORs). We identified 322 titles, reviewed 30 articles for inclusion and exclusion criteria, and included ten studies in the meta-analysis. The odds of pregnancy loss in women with FVL (absolute risk 4.2%) was 52% higher (OR = 1.52, 95% confidence interval [CI] 1.06–2.19) as compared with women without FVL (absolute risk 3.2%). There was no significant association between FVL and pre-eclampsia (OR = 1.23, 95% CI 0.89–1.70) or between FVL and SGA (OR = 1.0, 95% CI 0.80–1.25). PGM was not associated with pre-eclampsia (OR = 1.25, 95% CI 0.79–1.99) or SGA (OR 1.25, 95% CI 0.92–1.70).

**Conclusions:**

Women with FVL appear to be at a small absolute increased risk of late pregnancy loss. Women with FVL and PGM appear not to be at increased risk of pre-eclampsia or birth of SGA infants.

*Please see later in the article for the Editors' Summary*

## Abstract 2

Marc Rodger and colleagues report the results of their systematic review and meta-analysis of prospective cohort studies that estimated the association of maternal factor V Leiden and prothrombin gene mutation carrier status and placenta-mediated pregnancy complications.

## Editors' Summary

**Background:**

The death of a baby at any stage of pregnancy is heartbreaking and, sadly, a quarter of women lose their baby during pregnancy or birth. A pregnancy can go wrong for many reasons but complications that are caused by problems with the placenta affect more than one in 20 pregnancies. The placenta is the organ that links the mother to her baby. It is full of blood vessels that transfer oxygen and nutrients from the mother to her baby and that take carbon dioxide and waste products away from the baby. If the placenta does not circulate blood efficiently between the mother and baby (placental insufficiency), the result can be pregnancy loss (spontaneous miscarriage or still birth), pre-eclampsia (a sudden rise in blood pressure in late pregnancy that is life-threatening for both mother and baby), a small for gestational age pregnancy (the baby does not grow properly during pregnancy), or placental abruption (separation of the placenta from the wall of the womb, a condition that deprives the baby of oxygen and nutrients and can cause severe maternal blood loss).

**Why Was This Study Done?:**

One possible cause of placental insufficiency is inherited thrombophilia, an increased tendency to form blood clots that occurs in more than 10% of people. The commonest inherited thrombophilias are factor V Leiden (FVL) and prothrombin gene mutation (PGM). Comparisons of the frequencies of these thrombophilias in women who have had placenta-mediated pregnancy complications with the frequencies in women who have not had complications (“retrospective case control studies”) have found an association between thrombophilia and pregnancy complications. As a result, doctors sometimes give heparin to women with thrombophilia who have had a poor pregnancy outcome to reduce blood clotting during subsequent pregnancies (anticoagulant therapy). However, a better way to determine whether thrombophilia and pregnancy problems are associated is to recruit groups of women with and without thrombophilia and follow them during pregnancy to see whether they develop complications —“prospective cohort studies.” In this study, the researchers undertake a systematic review (a search that uses predefined criteria to identify all the research on a given topic) and meta-analysis (a statistical method for combining the results of studies) of prospective cohort studies to estimate the risk of placenta-mediated pregnancy complications in women with FVL or PGM.

**What Did the Researchers Do and Find?:**

The researchers identified ten prospective cohort studies that examined the association between FVL/PGM and placenta-mediated pregnancy complications and that met their predefined criteria. In their meta-analysis of these studies, they estimated that the absolute risk of pregnancy loss in women with FVL was 4.2% whereas the absolute risk of pregnancy loss in women without FVL was 3.2%. In other words, women with FVL had a 52% higher risk of pregnancy loss than women without FVL (an odds ratio of 1.52). The absolute increased risk, however, was 1%. There was no significant association (a significant association is one that is unlikely to have occurred by chance) between PGM and pregnancy loss. Similarly, there was no significant association between either of the thrombophilias and pre-eclampsia, small for gestational age pregnancies, or placental abruption. Finally, there was no significant association between either FVL or PGM and the composite outcome of any placenta-mediated pregnancy complication (pregnancy loss, pre-eclampsia, small for gestational age, and placental abruption).

**What Do These Findings Mean?:**

These findings suggest that women with FVL have a small absolute increased risk of pregnancy loss but that neither FVL nor PGM increase a woman's risk of pre-eclampsia or of giving birth to a small for gestational age infant. Although there seems to be no increased risk of pregnancy loss with PGM, more research is needed to confirm this finding and to confirm the lack of an association between thrombophilia and placental abruption. The researchers also warn that all these reassuring findings should be treated cautiously because of variability between the studies in how complications were defined. Importantly, however, these findings suggest that the introduction of anticoagulant therapies for women with thrombophilia and a history of pregnancy complications on the basis of retrospective case control studies might have been premature. Anticoagulant therapy should be considered experimental, therefore, until controlled trials of the approach have been completed.

**Additional Information:**

Please access these Web sites via the online version of this summary at http://dx.doi.org/10.1371/journal.pmed.1000292.

- Womenshealth.gov, a US Department of Health and Human Services resource, provides information on pregnancy complications
- Tommys, a UK charity that funds scientific research into the causes and prevention of miscarriage, premature birth, and stillbirth, has information on problems in pregnancy
- The March of Dimes Foundation, a nonprofit organization for pregnancy and baby health, also has information on complications during pregnancy, including a fact sheet on thrombophilias and pregnancy
- The US National Alliance for Thrombosis and Thrombophilia has detailed information on thrombophilia and an article on the evolving story of thrombophilia and pregnancy outcomes

## Introduction

Pregnancy loss, pre-eclampsia, small for gestational age (SGA) pregnancies, and placental abruption are distressing and often devastating pregnancy outcomes for women, their families, and society [1]–[3]. Frequently occurring as a result of placental insufficiency, they affect more than one in 20 pregnancies [3],[4]. Thrombophilia describes an increased tendency to develop thrombosis, either venous or arterial. Thrombophilias may either be inherited or acquired and include protein C deficiency, protein S deficiency, antithrombin deficiency, and the less potent factor V Leiden (FVL) and prothrombin gene mutation (PGM). The combined prevalence of these thrombophilias in the general population exceeds one in ten [5]–[11]. A successful pregnancy requires the development of adequate placental circulation. It is hypothesised that thrombophilias may increase the risk of placental insufficiency because of placental micro- and/or macro-vascular thrombosis, as well as effects on trophoblast growth and differentiation [12].

Individual case control studies variably demonstrate an association between the placenta-mediated pregnancy complications and thrombophilia but when meta-analysed suggest a weak positive association [13]–[15]. Case control studies are limited by (1) retrospective data collection leading to potential bias in outcome classification and incomplete and/or poor confounder data acquisition and (2) possible differential participation bias where more severe cases are recruited. Prospective cohort studies limit these potential biases but have limited power to detect weak associations [16]. Prospective cohort studies also provide absolute risk estimates that can be used to counsel patients. Our objective was to undertake a systematic review and a meta-analysis of prospective cohort studies to estimate the risk of these common and important placenta-mediated pregnancy complications in the women with and without FVL or PGM.

## Methods

### Search Strategy

Following our systematic review protocol, a systematic literature search strategy was conducted to identify potential studies in MEDLINE (1950 to November 2007) and EMBASE (1980 to November 2007) using the OVID interface. The search was updated in February 2010.We identified the following relevant MeSH and free terms for the exposures and outcomes by literature review and by recommendations from experts in the field (MAR, MC): Thrombophilia, activated protein C resistance (APCR), FVL, and PGM (PGM, PGV, G202110A, G1691A) for the exposure. For the outcome we used the subject headings pregnancy complications, abruption placentae, abortion spontaneous, stillbirth, pre-eclampsia, HELLP syndrome, hypertension-pregnancy induced, fetal growth retardation, low birth weight, and the free term miscarriage. Different subject headings were used for EMBASE or MEDLINE when appropriate. These searches were limited to observational studies by applying validated filters for MEDLINE and EMBASE available in the Scottish Intercollegiate Guidelines Network [17]. The search was also restricted to humans. There were no restrictions on language, publication year, or type of publication. Our full systematic search strategy is documented in Table S1. In addition to the electronic search, we examined reference lists of retrieved articles and identified additional articles and abstracts from recommendations of experts in the field. Duplicate reports of the same cohort reporting the same outcomes with the same exposures were excluded. Records of selected articles were examined by two independent reviewers (MTB and MAR) to identify all relevant citations.

### Selection Criteria

Using a structured question format to aid our literature search strategy, we reviewed all potentially relevant articles that satisfied all of the following criteria: (1) unselected pregnant women enrolled prospectively in the first or second trimester of pregnancy; (2) women investigated for FVL (diagnosed by DNA-based PCR assay for FVL mutation) or PGM (by DNA-based PCR assay) carrier status (homozygotes, heterozygotes, or both); and (3) reported any of the following placenta-mediated pregnancy complication outcomes: pregnancy loss, pre-eclampsia, placenta abruption, or SGA.

### Outcome Measures

The primary outcome measure was the incidence of placenta-mediated complications during pregnancy (pregnancy loss, pre-eclampsia, SGA, and placental abruption). Pre-eclampsia was defined as systolic blood pressure of 140 mm Hg or higher or a diastolic blood pressure of 90 mm Hg or higher occurring after 20 wk of gestation in a woman whose blood pressure has previously been normal; plus proteinuria, with excretion of 0.3 g or more of protein in a 24-h urine specimen or proteinuria ≥2 by dipstick [18],[19]. Pregnancy loss included (1) spontaneous miscarriage (involuntary termination of pregnancy before 20 wk of gestation, dated from the last menstrual period) or below a fetal weight of 500 g [20],[21] and (2) stillbirth (complete expulsion or extraction of a dead fetus at or after 20 wk of pregnancy, or when the fetal weight was at least 500 g in cases where the gestational age is not known) [22]. Placental abruption was defined as ante partum retroplacental, marginal, or preplacental hemorrhage confirmed by imaging studies or visual inspection of the placenta [23]. SGA was defined as a birth weight less than 10th percentile of population-specific birth weight adjusted for gender and gestational age. Severe SGA was defined as a birth weight less than 5th percentile of population-specific gender and gestational age-adjusted birth weight [24],[25].

### Data Extraction and Quality Assessment

Two reviewers (MTB and MAR) independently applied the inclusion criteria to the identified articles from the initial search strategy. Articles for potential full review were discussed between the two reviewers. A data-extraction form was designed, piloted, and revised. Reviewers independently extracted baseline characteristics of the included studies and obtained a 2×2 table with the number of placenta-mediated complications in exposed and unexposed patients (i.e., FVL or PGM status). The corresponding authors of studies with missing data were contacted. Discrepancies were noted and discussed between reviewers. Adjudication by a third party to resolve conflicts was not necessary.

The methodological quality of the studies was evaluated using the validated Newcastle–Ottawa scale (NOS) for prospective cohort studies as recommended by the Cochrane Non-Randomized Studies Methods Working Group [26]. The quality of a study was judged on the selection of the study groups, the comparability of the groups, and the ascertainment of the outcome of interest. For all eligible studies, two reviewers (MTB and MAR) independently assessed study quality and extracted the data using a standardized data abstraction form. Likewise, any discrepancies were documented and discussed between the reviewers.

### Data Synthesis and Analysis

We chose to use the pooled estimate of the odds ratio (OR) as our measure of effect, to analyze the results based on 95% confidence intervals (CIs), and to report two-sided *p*-values. Statistical heterogeneity between and within groups was measured using *I* ^2^ statistic [27]. *I* ^2^<25% was considered low heterogeneity, 25%–50% was moderate heterogeneity, and >50% was considered high heterogeneity [28]. We used Mantel-Haenszel analysis with 0.5 zero cell replacement method for this meta-analysis given event rates over 1% for the placenta-mediated pregnancy complications, imbalance in exposed/nonexposed groups, and imbalance in cases and controls [29]. In sensitivity analyses Peto ORs were also determined. Fixed-effect method was used to pool results for all models. We used sensitivity analysis to explore the causes of heterogeneity and to determine the impact that differences in outcome definition or measurement had on our results [30]. We also planned to examine funnel plots to assess publication bias if sufficient number of studies were included to permit assessment of publication bias.

The analysis was conducted using SAS 9.1 and RevMan 5.0 software from the Cochrane reviews.

## Results

**Figure 1:**
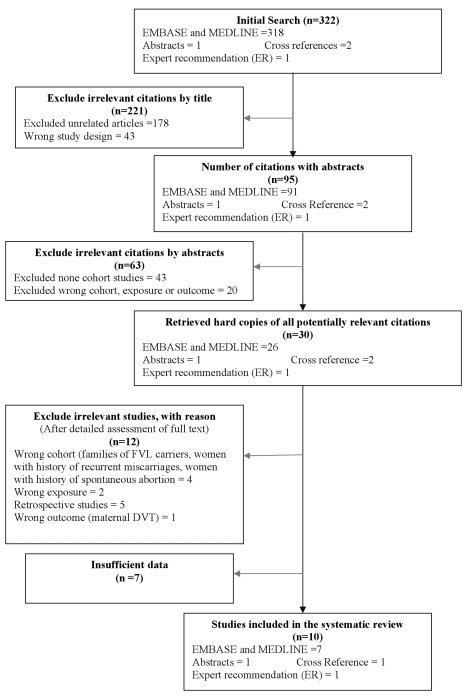
Study selection.

A total of 322 articles and one abstract (see Figure 1 for study selection flowchart) were identified. Of these, 95 were deemed relevant on the basis of their title. After abstract review, 30 articles and one abstract were selected for full article review or direct data retrieval from the authors. Of the 30 studies, 12 studies did not meet the inclusion criteria, and eight studies did not have sufficient data for inclusion after full article review. After contacting authors, we obtained data on the number of events and study design features for one of the eight published articles with insufficient data for this review [31]. We also obtained complete data for the abstract [32]. After reviewing the full text of the papers and contacting the authors we included ten articles in the review [31]–[40]. We did not exclude any study on the basis of the definition of outcome used by the authors.

### Included Studies Characteristics

The association between thrombophilias (FVL or PGM) and placenta-mediated pregnancy complications was summarized from ten prospective cohort studies. Of the included studies, there were cohorts from Dublin, Ireland [38]; Tel Aviv, Israel [31]; multiple centers in the United States, which gave rise to two publications, one reporting on FVL thrombophilia [34] and one reporting on PGM thrombophilia [40]; Glasgow, UK [33]; Avon, UK [35]; Melbourne, Australia [39]; Patras, Greece [36]; Malmo, Sweden [37] and Ottawa, Canada [32].

The characteristics of the selected studies are summarized in Table 1 and Table S2. The cohorts were homogeneous and consistent in terms of participants, exposures, and outcomes except for the outcomes of pregnancy loss and placental abruption. The latter definitions were either unclear and/or inconsistent across studies. Participants were women with spontaneous singleton pregnancy in either their first or second trimester except for one study where all patients were enrolled before 8 wk of gestation [36] and one study where a small number of multiple pregnancies were included [39]. Gestational age at enrollment ranged from 6 to 22 wk and most women were under 35 y of age. We were not able to find information on gestational age at enrollment for one study [35]. FVL prevalence (either homozygous or heterozygote) varied across selected studies from 2.7% [38] to 10.9% [37].

**Table 1:** Characteristics of included studies.

Study	City or State, Country	Gestational Age at Enrollment	Type of Thrombophilia and Prevalence, %, (*n*/Total *n*)	Study Population (Parity, Mean Age)	Outcome	Quality Assessment (NOS)
Silver et al., 2010 [40]	Maryland, United States	14 wk or less	PGM (+/− or ++), 3.8%, (156/4,167)	Mean age 25 (21–29) y	PET, SGA, PA, PL	Selection, ****; comparability, **; outcome, ***;
Said et al., 2010 [39]	Melbourne, Australia	Prior to 22 wk	FVL (+/− or ++), 5.4%, (93/1,726) and PGM (+/− or ++), 2.4%, (41/1,726)	100% nulliparous women, mean age 29.2±4.8 y	PET, SGA, PA, PL	selection, ****; comparability, *; outcome, **
Clark et al., 2008 [33]	Glasgow, UK	Range 7–16 wk	FVL(+/− or ++), 6.6%, (142/3,944)	45% primigravid women, mean age 28±6 y	PET, SGA, PL,	Selection, ****; comparability, **; outcome, ***
Dudding et al., 2008 [35]	Avon, UK	Unclear	FVL (+/− or ++), 7.5%, (587/7,869) and PGM (+/− or ++), 7.5%, (591/7,842)	44.3% nulliparous women, 98.7% <39 y	PET, SGA	Selection, ****; comparability, **; outcome, *
Karakantza et al., 2008 [36]	Patras, Greece	Range 6–8 wk	FVL (+/−), 3.3%, (13/392) and PGM (+/−), 3·1%, (12/392)	39.8% nulliparous women, 80.4% ≤35 y	PET, SGA, PA, PL	Selection, ****; comparability, 0; outcome, **
Rodger et al., 2007 [32]	Ottawa, Canada	Under 16 wk	FVL (+/− or ++), 4.5% (133/2,966) and PGM (+/− or ++), 2.1%, (63/2,939)	34.9% nulliparous women, mean age 31 y	PET, SGA, PA, PL	Selection, ****; comparability, *; outcome, *
Lindqvist et al., 2006 [37]	Malmo, Sweden	Mean 12 wk	FVL (+/− or ++), 10.9%, (270/2,480)	∼45% nulliparous women, mean age 29.2±4.7 y	PET, SGA, PA, PL	Selection, ****; comparability, **; outcome, ***
Dizon-Townson et al., 2005 [34]	Maryland, United States	14 wk or less	FVL (+/− or ++), 2.7%, (134/4,885)	30.7% primigravid women, mean age 25.8 (±5.6 y)	PET, SGA, PA, PL	Selection, ****; comparability, **; outcome, ***
Salomon et al., 2004 [31]	Tel Aviv, Israel	Range 14–16 wk	FVL (+/− or ++), 5.9%, (38/643) and PGM (+/− or ++), 6.2%, (40/643)	100% nulliparous women, mean age 28±3.3 y	PET, SGA, PA	Selection, ****; comparability, *; outcome, **
Murphy et al., 2000 [38]	Dublin, Ireland	Mean 14.2±0.26 wk	FVL (+/−), 2.7%, (16/588)	100% primigravid women, mean age 25±0.2 y	PET, SGA	Selection, ***; comparability, *; outcome, **

Abbreviations: +/−, heterozygous carrier; ++, homozygous carrier; NOS, Newcastle Ottawa Scale for cohort studies (maximum *n* of starts: selection, 4; compatibility, 2; outcome, 3); PA, placental abruption; PET, pre-eclampsia; PL, pregnancy loss.

The quality of the included studies as assessed using the Newcastle–Ottawa quality assessment scales scored high in selection and comparability of the study groups as well as in ascertainment of the outcome of interest (see Table 1).

The limited number of studies included in our meta-analysis did not permit assessment of publication bias.

### Maternal FVL/PGM and Pregnancy Loss

**Figure 2:**
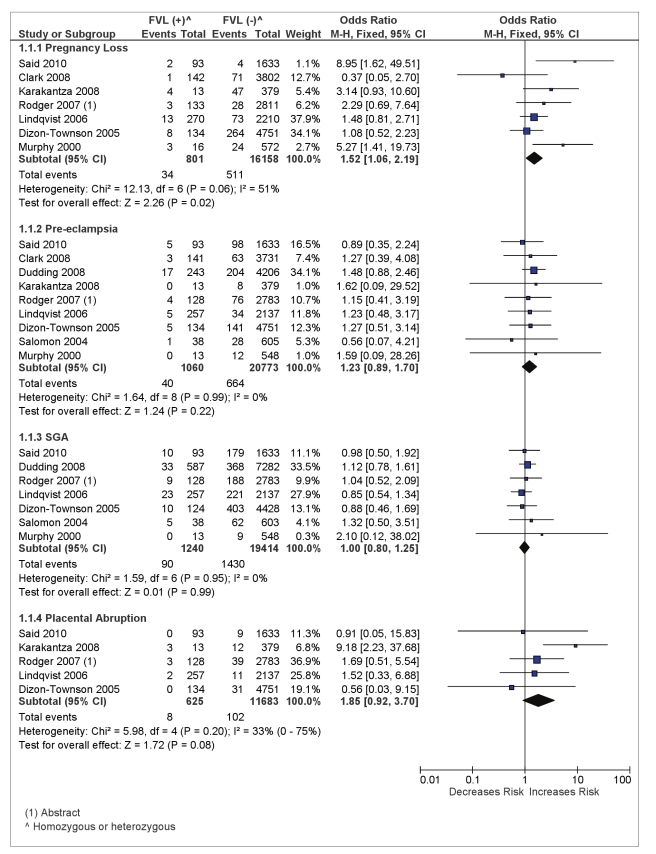
Odds of placenta-mediated pregnancy complications in FVL (homozygous or heterozygous)-positive women.

We identified seven studies reporting information on pregnancy loss [32]–[34],[36]–[39]. Most studies included patients with spontaneous miscarriage or stillbirth as pregnancy losses but there were important inconsistencies in the definition of this outcome. The pooled OR estimate from these seven studies is 1.52 (95% CI 1.06–2.19) in 16,959 total women with an observed FVL prevalence of 4.7% (see Figure 2). The absolute risk of pregnancy loss in women with FVL was 4.2% as compared with 3.2% for FVL negative women.

A fixed effect model was used for assessment of pregnancy loss despite substantial statistical heterogeneity across studies (*I* ^2^ = 51%, *p* = 0.06). The pooled estimate remained statistically significant when a random effect model was used 1.96 (95% CI 1.13–3.38) or when we used a Peto OR fixed model 1.64 (1.07–2.51). We suspected that differences in the definition of pregnancy loss across studies was the most likely explanation for this heterogeneity (see Table S2). Heterogeneity was significantly reduced when the two studies that included either spontaneous abortion only [38] or stillbirth only [39] were removed from the pooled analysis (*I* ^2^ = 15%, *p* = 0.32). After removing these two studies the pooled OR was no longer significant at 1.34 (95% CI 0.90–1.98). In addition, most studies enrolled both primiparous or multiparous women except the aforementioned two studies [38],[39], which included primiparous women only.

**Figure 3:**
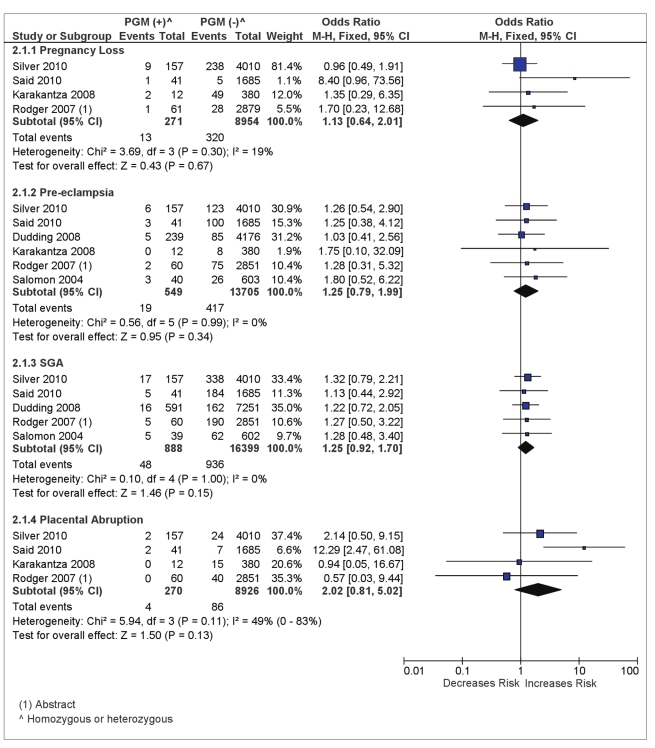
Odds of placenta-mediated pregnancy complications in PGM (homozygous or heterozygous)-positive women.

There were four studies reporting on the association of PGM and pregnancy loss with a pooled OR estimate of 1.13 and wide 95% CIs (0.64–2.01) (see Figure 3).

### Maternal FVL/PGM and Pre-eclampsia

All ten selected studies reported information on pre-eclampsia. The presence of FVL mutation did not significantly increase the risk of pre-eclampsia, with a pooled OR estimate of 1.23 (95% CI 0.89–1.70) in 21,833 total women with a FVL prevalence of 4.9% (see Figure 2). The absolute risk of pre-eclampsia in FVL positive women was 3.8% as compared with 3.2% for FVL-negative women. There was no statistical heterogeneity across studies and the definition of the outcome pre-eclampsia was fairly consistent across the studies (see Table S2).

The combination of six studies reporting on PGM status and pre-eclampsia did not show significant association between PGM (heterozygous or homozygous) and pre-eclampsia with a pooled OR = 1.25 (95% CI 0.79–1.99) in 14,254 total women with a PGM prevalence of 4.1%.(see Figure 3). The absolute risk of pre-eclampsia in women with PGM was 3.5% as compared with 3.0% for PGM negative women.

### Maternal FVL/PGM and SGA Neonate (Birth Weight <10th Percentile)

There were seven studies reporting information on SGA and FVL [31],[32],[34],[35],[37]–[39]. All studies had a fairly homogeneous definition of SGA although some heterogeneity may have been introduced by three studies that used country-specific birth-weight standardized charts (see Table S2) [32],[35],[39]. The presence of FVL did not significantly increase the risk of SGA, with a pooled OR estimate of 1.0 (95% CI 0.80–1.25) in 20,654 total women with a FVL prevalence of 6.0% (see Figure 3). The absolute risk of SGA <10th percentile in women with FVL was 6.5% as compared with 7.4% for FVL-negative women. This lack of association did not vary after exclusion of the three studies that used country-specific birth-weight charts (OR = 0.91, 95% CI 0.64–1.28) [32],[35],[39].

Five studies reported PGM status and SGA <10th percentile [31],[32],[35],[39],[40] in 17,287 total women with a PGM prevalence of 5.1%. The absolute risk of SGA <10th percentile in women with PGM (heterozygous or homozygous) was 5.4% as compared with 5.7% for PGM negative women. We did not find a significant association between PGM (heterozygous or homozygous) and SGA <10th percentile (pooled OR = 1.25, 95% CI 0.92–1.70) (see Figure 3).

### Maternal FVL/PGM and Severe SGA Neonate (Birth Weight <5th Percentile)

Five studies reported FVL status and birth weight under 5th percentile [33],[34],[36],[37],[39],[41],[42] with a total of 12,936 women, FVL prevalence 4.9%. The absolute risk of SGA <5th percentile in women FVL positive was 3.8% as compared with 4.3% for women FVL negative. The combined OR for this population showed no significant association between FVL status and SGA <5th percentile (pooled OR = 0.92, 95% CI 0.61–1.40). The pooled OR estimate of three studies reporting on SGA <5th percentile and PGM was 1.46 (0.81–2.62) in 6,285 total women with a prevalence of 3.3% for PGM. The prevalence of SGA <5th percentile in women with PGM was 5.7% as compared with 4.3% for PGM negative women.

### Maternal FVL/PGM and Placental Abruption

There were five studies reporting the association between FVL mutation and placenta abruption [32],[34],[36],[37],[39]. These studies included 12,308 women with a pooled FVL prevalence of 5.1%. The absolute risk of placenta abruption in FVL positive women was 1.3% as compared with 0.9% for FVL negative women. The pooled OR estimate for placental abruption in women with FVL mutation (homozygous or heterozygous) was 1.85 (95% CI 0.92–3.70) (see Figure 2). The moderate statistical heterogeneity with *I* ^2^ = 33% may be attributable to the inconsistent and unclear definition of placental abruption across studies (see Table 1).

The pooled OR estimate for placental abruption in women with PGM mutation (homozygous or heterozygous) was 2.02 (95% CI 0.81–5.02) with moderate heterogeneity across studies (*I* ^2^ = 49%) (see Figure 3).

The results for all four individual outcomes were not substantially different in sensitivity analysis using Peto ORs.

### Maternal FVL/PGM and the Composite of Any Placenta-Mediated Pregnancy Complications

**Figure 4:**
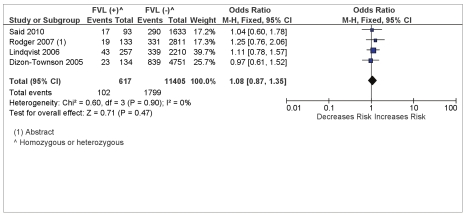
FVL and composite placenta-mediated pregnancy complications.

**Figure 5:**
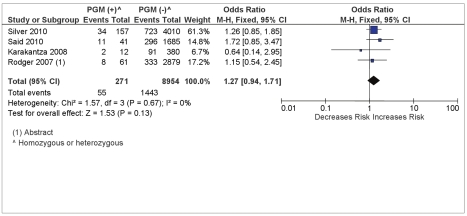
PGM and composite placenta-mediated pregnancy complications.

There was no association between FVL and the composite outcome of any of the placenta-mediated pregnancy complications (pregnancy loss, placental abruption, pre-eclampsia, and SGA [>10th percentile]) with a pooled OR = 1.08 (95% CI 0.87–1.52) from four studies reporting on the four outcomes (see Figure 4). There was no association between PGM and the composite outcome of any placenta-mediated pregnancy complications (pregnancy loss, placental abruption, pre-eclampsia, and SGA [>10^th^ percentile]) with a pooled OR = 1.27 (95% CI 0.94–1.71) from four studies reporting on the four outcomes (see Figure 5).

## Discussion

The principal findings of our meta-analysis of prospective cohort studies examining a potential association between FVL or PGM are (1) we have shown that FVL is likely weakly associated with pregnancy loss and (2) we have shown that neither FVL nor PGM are associated with pre-eclampsia or birth of an SGA infant. Further research is required to determine if FVL or PGM are associated with placental abruption and whether PGM is associated with important increases in pregnancy loss.

Pregnancy loss, especially recurrent or late pregnancy loss, is a painful event for pregnant women and their families [3]. Despite demonstrating that the odds of pregnancy loss in women with FVL appears to be 52% higher as compared with women without FVL, women with FVL should be reassured that the absolute event rate for pregnancy loss is low (4.2%) and only appears slightly higher than the rate of pregnancy loss in women without FVL (3.2%). As discussed below, this finding must be interpreted with caution given the statistical and clinical heterogeneity in this analysis. If a theoretical intervention was demonstrated to completely eliminate this increased risk of pregnancy loss in women with FVL (i.e., 100% relative risk reduction) it would result in a number needed to treat (NNT) of 100. That is 100 women would require treatment to prevent one pregnancy loss. Our findings are also in sharp contrast to the initial case control study reports of a strong association between FVL and pregnancy loss (e.g., ORs 4.9 with FVL and stillbirth) [41] but consistent with later meta-analyses [14].

We unfortunately had insufficient sample size to detect important increases in the risk of pregnancy loss in women with PGM. With a PGM prevalence of 2.9% in a sample size of 9,225, we only have 80% power to detect an absolute increase of >4% from the observed control group event rate of 3.6%. As such we had limited power to detect important differences in absolute risk of pregnancy loss (e.g., 1% or 2%) in women with PGM.

We found substantial statistical heterogeneity across studies reporting pregnancy loss. It is well known that nonuniform definitions of pregnancy loss across studies may create heterogeneity [42]. The likely explanations of the heterogeneity we observed in our meta-analysis include the wide range of gestational ages at enrollment (6 to 22 wk) in the component studies, the inconsistent definition of pregnancy loss across studies, and perhaps differences in parity in inclusion criteria between studies. Pregnancy loss was defined as any loss (i.e., spontaneous miscarriage or stillbirth) for most of the studies, but as spontaneous miscarriage only for one study [38], and as stillbirth only for one study [39]. Heterogeneity was significantly reduced when two studies including only primiparous women [38],[39] were removed from meta-analysis.

Pre-eclampsia is the most important cause of premature delivery with the resultant impact on fetal and neonatal morbidity and mortality [2]. Our study failed to demonstrate an association between the genetic thrombophilias and pre-eclampsia yet had excellent power to detect these associations. We had over 90% power to detect an absolute increase of 2% (from control 3.2% to FVL 3.2%+2% = 5.2%) in the rate of pre-eclampsia in women with FVL and an increase of 3% (from control 3.4% to PGM 3.4%+3.0% = 6.4%) in the rate of pre-eclampsia in women with PGM, yet we did not detect any increased risk. This finding should allow clinicians to provide reassurance to women with these thrombophilias that they are not at significantly increased risk of pre-eclampsia.

SGA often results in long-term effects in the developing child, including developmental delay and poor school performance and, as adults, children with SGA are significantly less likely to attain higher academic and professional achievement [43]. A recently published meta-analysis of case control and cohort studies identified a significant association between FVL and SGA (<10th percentile) in case control studies but identified evidence of publication bias in these case control studies [44]. In their analysis of retrospective and prospective cohort studies, there was no evidence of association between FVL and SGA (<10th percentile) (*n* = 3 studies with 8,256 patients), and the authors failed to identify any cohort studies examining an association between PGM and SGA [44]. Our meta-analysis, which only included prospective cohort studies, included more publications and more patients (FVL, *n* = 7 with >20,000 patients; PGM, *n* = 5 with >17,000 patients) as we included abstracts and we contacted the authors to obtain missing information, hence we can provide more robust and precise estimates of association. Our study failed to demonstrate an association between the genetic thrombophilias and SGA yet had excellent power to detect an association. We had over 90% power to detect an absolute increase of 3% (from control 7.4% to FVL 7.4%+3% = 11.4%) in the rate of SGA (<10th percentile) in women with FVL and an increase of 3% (from control 5.4% to PGM 5.4%+3% = 8.4%) in the rate of SGA (<10th percentile) in women with PGM, yet we did not detect any increased risk. This finding should allow clinicians to provide reassurance to women with these thrombophilias that they are not significantly more likely to give birth to an SGA child.

We had inadequate power to detect a doubling of risk of placental abruption in women with FVL or women with PGM. Unfortunately small sample sizes and low event rates limit conclusions regarding an association between the inherited thrombophilias, FVL or PGM, and placenta abruption. Furthermore, our analyses were limited by considerable statistical heterogeneity likely resulting from variable definitions for placental abruption.

The strengths of our study include: (1) the large number of pregnancies collectively examined in prospective cohort studies, which allows us to detect/exclude relatively small effects as outlined above; (2) the inclusion of only prospective cohort studies, which allows for more accurate directed data collection that minimised outcome misclassification and likely improves completeness and accuracy of confounder data collection; (3) prospective design that allows participant recruitment prior to outcome determination thereby minimising selection bias; (4) we suspect that prospective cohort studies are less likely than case control studies to suffer from publication bias, as explained below; and (5) the combination of prospective data without heterogeneity from different parts of the western world increases the generalizability of the study.

The limitations of our meta-analysis include: (1) prospective cohort studies enrolling pregnant patients beyond the late first trimester do not permit examination of early pregnancy events, such as early pregnancy loss, prior to enrollment. Only one out of seven studies reporting information on pregnancy loss exclusively enrolled patients early in their first trimester of pregnancy [36]. Hence, we could not examine for an association with early pregnancy loss. Only “conception” or prepregnancy prospective cohorts would permit prospective examination of this issue; (2) we had insufficient data and hence power to detect important associations between PGM and pregnancy loss as well as FVL or PGM and placental abruption; (3) there are insufficient prospective cohort studies examining the less common and more potent thrombophilias such as antithrombin, protein C, and protein S deficiencies to elucidate associations between these thrombophilias and placenta-mediated pregnancy complications. Given the expense of biological assays for these thrombophilias, the need for repeat confirmatory testing and false positives with protein S in pregnancy it is unlikely that adequately powered prospective cohort studies will be conducted to address the question of an association between these thrombophilias and placental-mediated pregnancy complications.

Our findings are in sharp contrast to the initial case control study reports of a strong association between thrombophilia and pre-eclampsia and SGA [43], and in contrast to later meta-analyses of case control studies [13],[15],[16],[41]. Several explanations are possible for the discrepancy between case control and prospective cohort studies in this area: (1) Publication bias: given the tremendous expense and effort in completing prospective cohort studies, negative studies are probably more likely to be published than small inexpensive negative case control studies. If this is the case, then proportionately more positive case control studies may be published and lead to the discrepant findings, indeed meta-analyses of case control studies in this area have suggested publication bias [44],[45]. The small number of cohort studies included in our analysis of prospective cohort studies limits the ability to detect publication bias in these prospective cohort studies; (2) Interaction: it may be that thrombophilias do not cause pre-eclampsia and SGA but synergise additional risk factors for these complications (i.e., have an interactive effect). Given that selection bias in case control studies may lead to more severe cases being included than in cohort studies, interactive effects may be more easily detected in case control studies and associations with thrombophilia may be confounded by additional risk factors that are more likely in selected case control studies.

The negative findings described in this publication are important. The small step of previously describing an association in case control studies has led a number of clinicians and opinion leaders to take the large leap of accepting this relationship as being causal and potentially treatable with anticoagulant interventions. Many have adopted low molecular weight heparin as part of routine care for women with a history of placenta-mediated complications who have tested positive for a laboratory marker of thrombophilia [1],[46],[47], and some have even adopted this practice in women with prior placenta-mediated pregnancy complications without thrombophilia, on the presumption that they harbour yet to be discovered thrombophilia. However, recent randomised trials have shown that low molecular weight heparin in unselected women with prior recurrent early loss do not benefit from low molecular weight heparin [48],[49]. Further trials are needed to determine if low molecular weight heparin will be of benefit in thrombophilic women with prior placenta-mediated pregnancy complications and in unselected women with the other placenta-mediated pregnancy complications (late loss, SGA, pre-eclampsia, abruption). Our findings highlight that in the absence of “no intervention” controlled studies, adopting anticoagulant prophylaxis to prevent these complications is premature and should be considered experimental.

In summary, women with FVL appear to be at a small absolute increased risk of late pregnancy loss. Women with FVL and PGM appear not to be at increased risk of pre-eclampsia or birth of SGA infants. Further research is required to determine if PGM is associated with pregnancy loss and whether FVL or PGM are associated with placental abruption. Adopting anticoagulant prophylaxis to prevent these complications in thrombophilic women is premature and should be considered experimental.

## Supporting Information

Checklist S1 PRISMA 2009 checklist.
(0.07 MB DOC)

Protocol S1 The association of FVL and PGM and placenta-mediated pregnancy complications.
(0.16 MB DOC)

Table S1 Search strategy.
(0.03 MB DOC)

Table S2 Outcome definition/adjudication.
(0.08 MB DOC)

## Abbreviations

CI: confidence interval
FVL: factor V Leiden
PA: placental abruption
PET: pre-eclampsia
PGM: prothrombin gene mutation
PL: pregnancy loss
OR: odds ratio
SGA: small for gestational age
