# Supplementary material for: The Association of Factor V Leiden and Prothrombin Gene Mutation and Placenta-Mediated Pregnancy Complications: A Systematic Review and Meta-analysis of Prospective Cohort Studies
Source: PLoS Med. 2010 Jun 15;7(6):e1000292. doi: 10.1371/journal.pmed.1000292 (PMC2885985; doi:10.1371/journal.pmed.1000292)
Supplement: Protocol S1 — The association of FVL and PGM and placenta-mediated pregnancy complications. (0.16 MB DOC) [file pmed.1000292.s002.doc]

**PICOS Question**

In pregnant women, does FVL or PGM carrier status affects the risk of placenta mediated pregnancy complications (pregnancy loss, pre-eclampsia, placenta abruption, and intrauterine growth restriction (IUGR))? A systematic review of prospective cohort studies that recruit pregnant women in early pregnancy, assesses their FVL or PGM carrier status, follows them up until delivery to compare the risk of placenta mediated pregnancy complications among women with exposure (FVL or PGM carrier) and those without.

***Do pregnant women with Factor V Leiden or PGM mutations have a high risk of placenta- mediated pregnancy complications?***

| Explanation | | |
| --- | --- | --- |
| P | Population | *Pregnant women* |
| I | Exposure (Intervention under examination) | *FVL - PGM + TEST* |
| C | Comparison intervention | *FVL - PGM - TEST* |
| O | Outcomes | *Placenta-mediated pregnancy complications:*  *Pregnancy loss*  *Preeclamsia*  *Placental abruption*  *Intrauterine growth restriction* |
| S | Study design | *Prospective cohort studies* |

**Objectives**

The primary objective of this review is to combine data available from prospective cohort studies of Factor V Leiden or Prothrombin Gene Mutation carriers in pregnant women to determine the association of these thrombophilias with placenta mediated pregnancy complications

**Criteria for Considering Studies for This Review**

Type of studies:

We will include all studies that prospectively follow pregnant women.

Type of participants:

Pregnant women enrolled at early pregnancy (first or second trimester of pregnancy).

Type of exposure:

Studies that reported on Factor V Leiden or Prothrombin Gene Mutation carrier status (homozygous, heterozygous or both). APCR and genetic testing included?

Presence of homozygosity or heterozygosity for Factor V Leiden diagnosed by DNA-based test for factor V mutation; or Prothombin Gene Mutation defined as the substitution of glutamine for arginine at position 20210 in the 3’ untranslated region of the prothrombin gene .

Type of outcome measure:

Studies that reported on at least one of the following placenta mediated pregnancy complication outcomes:

*Pre-eclampsia:* defined as a systolic blood pressure of 140 mm Hg or higher or a diastolic blood pressure of 90 mm Hg or higher occurring after 20 weeks of gestation in a woman whose blood pressure has previously been normal; Proteinuria, with excretion of 0.3 g or more of protein in a 24-hour urine specimen or proteinuria >=2 by dipstick (Helewa et al., 1997;Schroeder and American College of Obstetricians and Gynecologists, 2002).

*Pregnancy loss:* Includes spontaneous abortion or stillbirth. Spontaneous abortion defined as the involuntary termination of pregnancy before 20 weeks of gestation (dated from the last menstrual period) or below a fetal weight of 500g. Stillbirth defined as losses after 20 weeks of gestation (Speroff and Fritz, 2005)

Placental abruption: Defined as a condition in which some part of the placenta prematurely separates from the uterus including retroplacental, marginal, or preplacental confirmed by imaging studies (Helen H, 2003).

Small for gestational age infants (SGA) defined as a birth weight that is more than two standard deviations below the mean distribution of normal population (birth weight less than 10^th^ percentile of a population specific birth weight versus gestational age plot). Severe SGA defined as a birth weight that is more than three standard deviations below the mean distribution of normal population (birth weight less than 5^th^ percentile of a population specific birth weight versus gestational age plot) (Anderson and Hay, 2005;Galan and Hobbins, 2003).

**Search Methods for Identification of Studies**

An electronically search of the following databases using an OVID interface will be performed: MEDLINE, EMBASE . Adjustments will be made to the search strategy to account for the differences in indexing between databases. There will be no restriction on language. These searches will be limited to observational studies by applying validated filters for Medline and Embase available in the Scottish Intercollegiate Guidelines Network (SIGN, 2008).

Complete references of all articles into RefWorks and duplicates will be removed within the RefWorks program.

The authors will search MEDLINE using the following terms (Appendix 1):

APCR (text word) or FVL (text word) or PGM (text word) or PGV (text word) or G20210A (text word) or G1691A (text word) or Thrombophilia (MeSH).

Pregnancy (MeSH) or placenta (MeSH) or pregnancy loss (text word) or fetal loss (text word) or embryonic loss (text word) or placental abruption/abruption placentae (text word) or IUGR/Intrauterine growth retardation/Intrauterine growth restriction (text word) or VLBW/very low birth weight (text word) or fetal growth retardation (text word) or miscarriage (text word) or Fetal death/Stillbirth (MeSH) or pre-eclampsia (MeSH) or high blood pressure (text word) or HELLP Syndrome (MeSH) or neonatal morbidity (text word) or maternal morbidity (text word).

**Methods of the Review**

Selection of prospective cohort studies

Two authors will independently screen the title and abstract of identified records for potential eligibility. Full text of articles judged potentially eligible will be retrieved. Discrepancies will be resolved by discussion between reviewers.

The two authors will then independently assess the full text of the selected article for inclusion into the present review. Any disagreements will be resolved by consulting a third party. In order to be included the trial will have to meet the following criteria:

*Inclusion Criteria:*

1. Pregnant women at first or second trimester of pregnancy
2. Pregnat women were investigated for Factor V Leiden or Prothrombin Gene Mutation carrier status
3. Prospective cohort studies of consecutive patients
4. One or more placenta mediated pregnancy complication outcomes are reported

*Exclusion Criteria:*

1. Duplicated publications
2. Insufficient data for an effect measure of FVL or PGM and placenta related pregnancy complications.
3. Retrospective studies
4. Prospective studies not following consecutive patients

A flow diagram/chart will be used to tract included or excluded studies at each step of the search process.

Data extraction (Appendix 2)

Both authors will independently extract the data from the retrieved articles. The primary authors of any articles with inadequate or missing information will be contacted. Any discrepancies will be resolved by consensus.

Statistical analysis

The analysis will be performed separately for FVL and PGM carrier status. We will extract 2X2 tables with number of outcome events per exposed and unexposed pregnant women from each selected study. Pooled Relative Risk (RR) will be the measure of effect of choice. We will analyzed maternal FVL or PGM results in association with 4 separate placenta mediated pregnancy complications outcomes: a) Pregnancy loss: b) Pre-eclampsia c)Placental abruption and d) Intrauterine Growth Retardation (IUGR). 95% confidence intervals for these estimates will be calculated. Comparison between groups will be done using the X2 statistical methods using two-sided p values.

Statistical heterogeneity between and within groups will be measured using I2 statistic. An I2 of < 25% will be considered as low-level heterogeneity, 25% to 50% moderate level and higher than 50% high level. For statistically homogeneous data, RR pooled estimates will be calculated using a fixed effect model. For heterogeneous data, a random effect model will be used and subgroup analysis will be conducted to explore reasons.

The analysis will be conducted using SAS and RevMan 4.2 software from the Cochrane reviews.

**Potential Conflict of Interest**

None known

**Appendix 1. SEARCH STRATEGY**

Ovid MEDLINE(R)
1950 to November Week 2 2007/ updated on September 2008

| | **#** | **Search History** | | --- | --- | | 1 | Activated Protein C Resistance.mp. or Activated Protein C Resistance/ or Factor V/ | | 2 | FVL.mp. | | 3 | factor V leiden.mp. [mp=title, original title, abstract, name of substance word, subject heading word] | | 4 | prothrombin gene mutation.mp. [mp=title, original title, abstract, name of substance word, subject heading word] | | 5 | PGV.mp. [mp=title, original title, abstract, name of substance word, subject heading word] | | 6 | PGM.mp. [mp=title, original title, abstract, name of substance word, subject heading word] | | 7 | G20210A.mp. [mp=title, original title, abstract, name of substance word, subject heading word] | | 8 | G1691A.mp. [mp=title, original title, abstract, name of substance word, subject heading word] | | 9 | exp Thrombophilia/ | | 10 | or/1-9 | | 11 | Pregnancy/ or Pregnancy Outcome/ or Hypertension, Pregnancy-Induced/ | | 12 | Placenta/ | | 13 | pregnancy loss.mp. | | 14 | fetal loss.mp. [mp=title, original title, abstract, name of substance word, subject heading word] | | 15 | embryonic loss.mp. [mp=title, original title, abstract, name of substance word, subject heading word] | | 16 | placental abruption.mp. [mp=title, original title, abstract, name of substance word, subject heading word] | | 17 | abruption placentae.mp. [mp=title, original title, abstract, name of substance word, subject heading word] | | 18 | IUGR.mp. [mp=title, original title, abstract, name of substance word, subject heading word] | | 19 | VLBW.mp. [mp=title, original title, abstract, name of substance word, subject heading word] | | 20 | very low birth weight.mp. [mp=title, original title, abstract, name of substance word, subject heading word] | | 21 | fetal growth retard$.mp. [mp=title, original title, abstract, name of substance word, subject heading word] | | 22 | Intrauterine growth retard$.mp. [mp=title, original title, abstract, name of substance word, subject heading word] | | 23 | intrauterine growth restrict$.mp. [mp=title, original title, abstract, name of substance word, subject heading word] | | 24 | miscarriage.mp. or Abortion, Spontaneous/ | | 25 | Fetal Death/ or Stillbirth/ | | 26 | Pre-Eclampsia/ | | 27 | high blood pressure.mp. [mp=title, original title, abstract, name of substance word, subject heading word] | | 28 | HELLP Syndrome/ | | 29 | neonatal morbidity.mp. | | 30 | maternal morbidity.mp. [mp=title, original title, abstract, name of substance word, subject heading word] | | 31 | or/11-30 | | 32 | cohort studies/ or longitudinal studies/ | | 33 | (cohort adj (study or studies)).tw. | | 34 | Cohort analy$.tw. | | 35 | (Follow up adj (study or studies)).tw. | | 36 | (observational adj (study or studies)).tw. | | 37 | Prospective studies/ | | 38 | or/32-37 | | 39 | and/10,31,38 | |  |  | |
| --- | --- | --- | --- | --- | --- | --- | --- | --- | --- | --- | --- | --- | --- | --- | --- | --- | --- | --- | --- | --- | --- | --- | --- | --- | --- | --- | --- | --- | --- | --- | --- | --- | --- | --- | --- | --- | --- | --- | --- | --- | --- | --- | --- | --- | --- | --- | --- | --- | --- | --- | --- | --- | --- | --- | --- | --- | --- | --- | --- | --- | --- | --- | --- | --- | --- | --- | --- | --- | --- | --- | --- | --- | --- | --- | --- | --- | --- | --- | --- | --- | --- | --- |

**Appendix 2. Data Extraction Form**

| Reviewer information | | | | | | | | | | | |
| --- | --- | --- | --- | --- | --- | --- | --- | --- | --- | --- | --- |
| Name: | | | | | | | | | | | |
| Date of extraction: | | | | | | | | | | | |
| Information about study references and authors | | | | | | | | | | | |
| Title of article | | |  | | | | | | | | |
| Primary Author | | |  | | | | | | | | |
| Year of publication | | |  | | | | | | | | |
| Type of document:   - Full journal article - Published abstract - Conference proceeding | | |  | | | | | | | | |
| Journal | | |  | | | | | | | | |
| Database source | | |  | | | | | | | | |
| Language of publication | | |  | | | | | | | | |
| Study number | | |  | | | | | | | | |
| Data source, specify:   - All data extracted from article - Some data provided by authors - All data provided by authors | | |  | | | | | | | | |
| **Verification of study eligibility (based in inclusion and exclusion criteria)**  If the study meets inclusion criteria the extraction of the data will continue, otherwise the study will be excluded at this point. | | | | | | | | | | | |
| Instructions: Answer yes, no or unclear | | | | | | | | | |  | |
| 1. Type of participants:  Adults >18 years old  Pregnant women. | | | | | | | | | |  | |
| 2. The Exposure and Comparison (any of the following):   - Factor V Leiden (FVL) + vs. FVL- - PCR ^FVL+^ vs. PCR ^FVL-^ - Prothombin Gen mutation (PGM) vs. FVL - | | | | | | | | | |  | |
| 3. Outcome measures:  Incidence placenta-mediated pregnancy complications(any of the following):   - Pregnancy loss (PL) - Pre-eclampsia (PE) - Placenta abruption (PA) - Intrauterine growth restriction (IUGR), or fetal growth retardation (FGR), or small per gestational age (SGA). | | | | | | | | | |  | |
| 4. Type of study:  Prospective cohort study | | | | | | | | | |  | |
| **Generalizability –External validity** | | | | | | | | | | | |
| Participants characteristics | | | | | | | | | | | |
| Country | | | | |  | | | | | | |
| Setting | | | | |  | | | | | | |
| Age range (mean) | | | | |  | | | | | | |
| Ethnicity | | | | |  | | | | | | |
| Study inclusion criteria | | | | |  | | | | | | |
| Study exclusion criteria | | | | |  | | | | | | |
| Co morbid conditions (specify Yes or No) :  Cardiovascular diseases  Diabetes  Others, specify | | | | |  | | | | | | |
| Number of patients (specify number):   1. Number of participants enrolled 2. Number of participants analyzed 3. Number of withdrawals 4. Number of loss to follow-up | | | | |  | | | | | | |
| Exposure – Answer “Yes”, “No”, or “unclear” | | | | | | | | | | | |
| FVL/PCR heterozygote | | | | | |  | | | | | |
| FVL/PCR homozygote | | | | | |  | | | | | |
| PGM heterozygote | | | | | |  | | | | | |
| PGM homozygote | | | | | |  | | | | | |
| Other thrombophilias, specify | | | | | |  | | | | | |
| FVL or PGM study sample rates:   - +/- or +/+ FVL exp rate - +/- or +/+ PGM exp rate | | | | | |  | | | | | |
| FVL or/and PGV testing techniques, describe. | | | | | |  | | | | | |
| **Outcome Measures** | | | | | | | | | | | |
| Primary outcome, describe. | | | | | |  | | | | | |
| Secondary outcomes, describe. | | | | | |  | | | | | |
| Outcome assessor blinding: yes, no, unclear. | | | | | |  | | | | | |
| How were outcomes adjudicated?   - Expert adjudication Committee - Expert single person - Other, specify | | | | | |  | | | | | |
| Was the outcome adjudicator blinded to FVL or PGM status? Yes, no, unclear. | | | | | |  | | | | | |
| Outcome definition (for each outcome, describe the study definition of outcome or outcome subrogate) | | | | | | | | | | | |
| Pre-eclampsia | |  | | | | | | | | | |
| IUGR | |  | | | | | | | | | |
| Placenta abruption | |  | | | | | | | | | |
| Pregnancy loss | |  | | | | | | | | | |
| **Outcome n/N extraction** | | | | | | | | | | | |
| **FVL (n/N)** | | | | | | | | | | | |
|  | FVL+ | | | | | | | FVL- | | | |
|  | (n) | | | Total (N) | | | | (n) | Total (N) | | |
| Pre-eclampsia |  | | |  | | | |  |  | | |
| IUGR < PC 10 |  | | |  | | | |  |  | | |
| IUGR < PC 5 |  | | |  | | | |  |  | | |
| Placenta abruption |  | | |  | | | |  |  | | |
| Pregnancy loss |  | | |  | | | |  |  | | |
| **PGM (n/N)** | | | | | | | | | | | |
|  | PGM+ | | | | | | | PGM - | | | |
|  | (n) | | | Total (N) | | | | (n) | Total (N) | | |
| Pre-eclampsia |  | | |  | | | |  |  | | |
| IUGR < PC 10 |  | | |  | | | |  |  | | |
| IUGR < PC 5 |  | | |  | | | |  |  | | |
| Placenta abruption |  | | |  | | | |  |  | | |
| Pregnancy loss |  | | |  | | | |  |  | | |
| **Newcastle-Ottawa (NOS) - Quality Assessment Scale for Cohort Studies** <http://www.ohri.ca/programs/clinical_epidemiology/oxford.htm>  The maximum starts (★) that can be given are: 4 starts for “selection”, 2 starts for “compatibility”, and 3 starts for “outcome” as per the NOS manual for cohort studies provided. | | | | | | | | | | | |
| **Instructions** | | | | | | | **Study Result** | | | | **NOS ★ Total** |
| **Selection (4)**   - Representativeness of the exposed cohort (truly representative*, somewhat representative*, selected groups, no description). - Selection of the non exposed cohort (drawn from the same community as the exposed*, drawn from a different source, no description). - Ascertainment of exposure (secure record*, structured interview*, written self report, no description) - Demonstration that outcome was not present at the start of the study (yes*, no). | | | | | | |  | | | |  |
| **Compatibility(2), describe:**   - Comparability of cohorts on the basis of the design or analysis (study controls for “parity”*) - Study control for additional factors* (e.g. Age, ethnicity etc)) | | | | | | |  | | | |  |
| **Outcome(3)**   - Assessment outcome (independent blind assessment*, record linkage*, self report, no description) - Was follow-up long enough for outcomes to occur – until delivery (yes*, no) - Adequacy of follow-up cohorts (complete follow-up*, subjects lost to follow up unlikely to introduce bias*, follow-up rate < 95% and no description of those lost, no statement. | | | | | | |  | | | |  |
