# Supplementary material for: The Association of Factor V Leiden and Prothrombin Gene Mutation and Placenta-Mediated Pregnancy Complications: A Systematic Review and Meta-analysis of Prospective Cohort Studies
Source: PLoS Med. 2010 Jun 15;7(6):e1000292. doi: 10.1371/journal.pmed.1000292 (PMC2885985; doi:10.1371/journal.pmed.1000292)
Supplement: Table S1 — Search strategy. (0.03 MB DOC) [file pmed.1000292.s003.doc]

**Table S1. Search Strategy**

| FVL and pregnancy complications – MEDLINE/EMBASE  February 2010 –MEDLINE/EMBASE  1. Activated Protein C Resistance.mp. or Activated Protein C Resistance/ or Factor V/  2. APCR.mp.  3. factor V leiden.mp.  4. FVL.mp.  5. Prothrombin/ or prothrombin gene mutation.mp.  6. PGM.mp.  7. prothrombin gene variant.mp.  8. PGV.mp.  9. G20210A.mp.  10. G1691A.mp.  11. exp Thrombophilia/  12. or/1-11  13. Pregnancy Complications/ or Pregnancy Trimester, Second/ or Pregnancy Trimester, Third/ or Pregnancy Complications, Cardiovascular/ or Pregnancy Trimester, First/ or Pregnancy Complications, Hematologic/ or Pregnancy/ or Pregnancy Outcome/ or Hypertension, Pregnancy-Induced/ or Pregnancy Trimesters/  14. exp Placenta/ or Abruptio Placentae/ or placenta abruption.mp. or Placenta Diseases/  15. Embryo Loss/ or Abortion, Spontaneous/ or Fetal Death/ or Pregnancy loss.mp.  16. Abruptio Placentae/ or placenta$ abruption.mp. or Uterine Hemorrhage/  17. IUGR.mp. or Fetal Growth Retardation/  18. Intrauterine growth retardation.mp.  19. Infant, Low Birth Weight/ or VLBW.mp. or Infant, Very Low Birth Weight/  20. miscarriage.mp. or Abortion, Spontaneous/  21. Stillbirth/  22. Pre-Eclampsia/ or Eclampsia/  23. HELLP Syndrome/  24. Hypertension/  25. neonatal morbidity.mp.  26. Maternal morbidity.mp.  27. intrauterine growth restrict$.mp. [mp=title, original title, abstract, name of substance word, subject heading word]  28. or/13-27  29. cohort studies/ or longitudinal studies/  30. (cohort adj (study or studies)).tw.  31. Cohort analy$.tw.  32. (Follow up adj (study or studies)).tw.  33. (observational adj (study or studies)).tw.  34. Prospective studies/  35. Longitudinal.tw.  36. or/29-35  37. and/12,28,36 |
| --- |
