# Supplementary material for: The Association of Factor V Leiden and Prothrombin Gene Mutation and Placenta-Mediated Pregnancy Complications: A Systematic Review and Meta-analysis of Prospective Cohort Studies
Source: PLoS Med. 2010 Jun 15;7(6):e1000292. doi: 10.1371/journal.pmed.1000292 (PMC2885985; doi:10.1371/journal.pmed.1000292)
Supplement: Table S2 — Outcome definition/adjudication. (0.08 MB DOC) [file pmed.1000292.s004.doc]

**Table S2. Outcome Definition/Adjudication**

| **Study** | **Blinded assessment of outcome** | **Adjudication by committee** | **Outcome definition** |
| --- | --- | --- | --- |
| **Pregnancy loss** | | | |
| Silver et al., 2010 | Yes | Yes | First trimester spontaneous abortion, fetal death (loss 14 - 20 weeks gestation), and stillbirth (> 20 weeks of gestation). |
| Said et al, 2010 | Yes | No | Stillbirth only (birth of an infant at least 400g or at least 20 weeks gestation with no signs of life after birth) Miscarriages not included. |
| Clark et al, 2008 | Yes | No | Spontaneous abortion (loss < 24 weeks gestation)  Stillbirth (loss ≥ 24 weeks gestation) |
| Karakantza et al, 2008 | Unclear | Unclear | Spontaneous abortion (loss < 24 weeks gestation)  Stillbirth (loss ≥ 24 weeks gestation)] |
| Rodger et al, 2007 | Yes | Yes | Spontaneous abortion or stillbirth |
| Lindqvist et al, 2006 | Yes | Yes | Spontaneous abortion (first trimester fetal loss before 13 weeks of gestation; second trimester fetal loss between 13-27 weeks of gestation) or stillbirth (> 27 weeks gestation) |
| Dizon-Townson et al, 2005 | Yes | Yes | Spontaneous abortion (first trimester), or fetal death (second trimester) or stillbirth (third trimester) |
| Murphy et al, 2000 | Unclear | Unclear | Spontaneous abortion (miscarriage after first trimester of pregnancy) |
| **Pre-eclampsia** | | | |
| Silver et al., 2010 | Yes | Yes | Diastolic blood pressure > 90 mmHg on 2 occasions  Proteinuria: ≥ 300 mg/day, urinary protein/creatinine ratio > 0·35, at least 2 + by dipstick in previously normotensive normoproteinuric patients.  Women with incomplete findings for pre-eclampsia with pulmonary edema or thrombocytopenia, women with eclampsia or women with HELP syndrome. |
| Said et al, 2010 | Yes | No | Blood pressure of 140/90 mmHg on at least 2 occasions occurring after 20 weeks in a previous normotensive pregnancy. Includes HELP syndrome.  Proteinuria: ≥ 300 mg/day or ≥ 2+ proteinuria by dipstick. |
| Clark et al, 2008 | Yes | No | Diastolic blood pressure > 90 mmHg on 2 occasions  Proteinuria: ≥ 2 + by dipstick |
| Dudding et al., 2008 | Unclear | Unclear | Systolic blood pressure > 140 mmHg  Proteinuria: ≥ 300 mg/day |
| Karakantza et al, 2008 | Unclear | Unclear | Pre-eclampsia: Blood pressure above 140/90 mm Hg after 20 weeks of gestation, proteinuria of 300 mg/24 h or a persistent level of 30 mg/dL (1+ dipstick) in random urine samples. |
| Rodger et al, 2007 | Yes | Yes | Blood pressure of 140/90 mmHg on at least 2 occasions plus ≥ 2 + by dipstick or proteinuria ≥ 300 mg/day |
| Lindqvist et al, 2006 | Yes | Yes | Diastolic blood pressure > 90 mmHg on 2 occasions developed after 20 weeks of gestation in a previously normotensive pregnancy.  Proteinuria: ≥ 300 mg/day. |
| Dizon-Townson et al, 2005 | Yes | Yes | Diastolic blood pressure > 90 mmHg on 2 occasions  Proteinuria: ≥ 300 mg/day, urinary protein/creatinine ratio > 0·35, at least 2 + by dipstick in previously normotensive normoproteinuric patients.  Women with incomplete findings for pre-eclampsia with pulmonary edema or thrombocytopenia, women with eclampsia or women with HELP syndrome. |
| Salomon et al, 2004 | Yes | No | Blood pressure of 140/90 mmHg on at least 2 occasions.  Proteinuria: ≥ 300 mg/day during II or III trimester |
| Murphy et al, 2000 | Unclear | Unclear | Blood pressure of 140/90 mmHg on at least 2 occasions or a single diastolic reading ≥ 110 mmHg.  Proteinuria: ≥ 1 + by dipstick  Resolution of hypertension by 6 weeks postpartum |
| **SGA** | | | |
| Silver et al., 2010 | Yes | Yes | Birth weight < 10^th^ percentile and < 5^th^ percentile derived from sex- and race-specific growth curves. |
| Said et al, 2010 | Yes | No | Birth weight < the 10^th^ percentile for gender and gestational age defined according to Australian birth centile charts.  Subgroup birth weight < the 5^th^ percentile. |
| Clark et al, 2008 | Yes | No | Birth weight < the 5^th^ percentile normalized by gestation at birth, fetal sex and maternal parity. |
| Dudding et al., 2008 | Unclear | Unclear | Birth weight < the 10^th^ percentile on gender-specific UK population charts |
| Karakantza et al, 2008 | Unclear | Unclear | Birth weight below the 5th percentile for gestational age |
| Rodger et al, 2007 | Yes | Yes | Birth weight < the 10^th^ percentile adjusted for gender and gestational age define according to Canadian birth centile charts |
| Lindqvist et al, 2006 | Yes | No | Birth weight < the 10^th^ percentile in weight deviation from a term reference population.  Subgroup birth weight < the 5^th^ percentile |
| Dizon-Townson et al, 2005 | Yes | Yes | Birth weight < the 10^th^ percentile from gender and race specific growth curves  Subgroup birth weight < the 5^th^ percentile |
| Salomon et al, 2004 | Yes | No | Birth weight < the 10^th^ percentile or less of the entire study group |
| Murphy et al, 2000 | Unclear | Unclear | Birth weight < the 10^th^ percentile of gestational age |
| **Placental Abruption** | | | |
| Silver et al., 2010 | Yes | Yes | Clinical suspicion plus documentation of excessive antepartum bleeding, treatment with blood products, description of delivery of the placenta and/or confirmation by pathologic examination. |
| Said et al, 2010 | Yes | No | Uterine tenderness associated with concealed or revealed hemorrhage in the presence of fetal distress, or maternal shock, or maternal coagulopathy. |
| Karakantza et al, 2008 | Unclear | Unclear | Separation of the placenta from its site of implantation before the delivery of the fetus documented by ultrasound in all cases. |
| Rodger et al, 2007 | Yes | Yes | Objective retroplacental clot. |
| Lindqvist et al, 2006 | Yes | Yes | Clinical diagnosis of abruption placenta including blood loss, clinical status, and by old blood clots in the uterus at caesarean. |
|  |  |  |  |
| Dizon-Townson et al, 2005 | Yes | Yes | Clinical suspicion plus documentation of excessive antepartum bleeding, treatment with blood products, description of delivery and placenta, and/or confirmation by pathologic examination. |
| Salomon et al, 2004 | Yes | No | No definition of outcome found. |
